# Isolation, identification and antimicrobial profile of methicillin‐resistant *Staphylococcus aureus* from bovine mastitis in and around Adama, Central Ethiopia

**DOI:** 10.1002/vms3.902

**Published:** 2022-08-22

**Authors:** Muhabaw Yimana, Juhar Tesfaye

**Affiliations:** ^1^ Addis Ababa University College of Veterinary Medicine, Department of Veterinary public health Debrezeit Ethiopia; ^2^ Ethiopian Institute of Agricultural Research Werer Agricultural Research Center, Animal health research department Werer Ethiopia; ^3^ Sekota Agricultural Bureau, Animal health case team Sekota Ethiopia

**Keywords:** Adama, antibiotic sensitivity, CMT, MRSA, oxacillin, resistance, risk factors

## Abstract

**Background:**

Among bacterial pathogens, *Staphylococcus aureus* which lives in the mammary gland is the leading cause of bovine mastitis worldwide, which causes enormous economic losses to the dairy industry.

**Objectives and methods:**

The study was carried out cross‐sectionally to determine the occurrence of methicillin‐resistant *S. aureus* (MRSA) and the risk factors for mastitis infection in dairy cows in and around Adama from October 2014 to June 2015. This particular study included 384 animals. Milk samples were collected and screened with California mastitis test. Then bacteria were cultured and identified using biochemical tests and disc diffusion test was used to determine the antimicrobial sensitivity for MRSA.

**Results:**

The prevalence of mastitis was 121 (31.5%). Among them, 37 cases (30.6%) were clinical mastitis and 84 cases (69.4%) were subclinical mastitis. Among these positive cases, 37 cases (30.6%) of *S. aureus* were isolated. The prevalence of mastitis was significantly related to the breed, age, floor type and sanitation status of the milking (*p* <0.05). The Kirby–Bauer disc diffusion method was performed on Mueller Hinton agar medium according to NCCLS guidelines to test antibiotic sensitivity. The 32.4% of *S. aureus* isolates were resistant to oxacillin. Isolates of MRSA are more resistant to amoxicillin (75%), oxytetracycline (66.7%) and sulfa (50%). However, they were sensitive to kanamycin (75%), streptomycin (58.3%) and nalidixic acid (50%). Insufficient dosage, short treatment time and chronic infection in dairy cows in the herd are the main reasons for the large number of resistant strains.

**Conclusion and recommendation:**

Generally, mastitis was prevalent in the area, and it was resistant to commonly used antibiotics. Therefore, hygienic, prevention and alternative treatment methods should be implemented.

## 1 INTRODUCTION

Milk produced by dairy cows is the food source for most rural and urban populations. The development of the Ethiopian dairy industry has helped to alleviate poverty and food insecurity in the country (Mohamed et al., 2004). However, this is limited by different reasons. Regardless of the animal species, mastitis can lead to reduced milk production and remains one of the most economically important diseases in the global dairy industry (Bradley, 2002). Mastitis can cause a decrease in the quality and quantity of milk (Quinn et al., 1999). Mastitis is distributed in dairy cows globally and has been described as of extreme economic and zoonotic importance (Al‐Majali et al., 2008). It is defined as clinical or subclinical and is a complex multifactorial disease. Its occurrence depends on different variables related to the host, the environment and the agent (Radostits et al., 2007).

Bacteria are the most common causes of mastitis which are found in the dairy cow and in the environment and are therefore a common threat to the mammary gland (Bradley, 2002). Among bacterial pathogens, *Staphylococcus aureus* is the leading cause of contagious bovine mastitis worldwide and it causes enormous economic losses to the dairy industry. Many species of staphylococci can produce a layer of extracellular polysaccharide and mucus, which are related to the virulence of the pathogen towards the host's defence mechanism. Other Non‐aures staphylococci are often found colonizing the teats and mammary glands. They rarely cause clinical mastitis and are called minor pathogens (Piepers et al., 2007; Radostits et al., 2007).

In addition to treatment, antibiotics are used in veterinary medicine for preventive purposes, to improve feed efficiency and promote growth particularly in developing countries. The large use of antimicrobials causes the emergence of antimicrobial‐resistant pathogens. Due to the emergence and spread of multidrug‐resistant zoonotic pathogens, the public and scientific communities are increasingly concerned about the widespread use of antimicrobial agents. Antibiotic‐resistant bacteria do not respond to conventional antibiotic treatment and prolongs the course of the disease. The resistance of *S. aureus* to antimicrobial agents can complicate the treatment of infections (Normanno et al., 2007). Shortly after the introduction of penicillin, around 1945, most *S. aureus* populations became resistant to penicillin by producing β lactamase (an enzyme that hydrolyzes penicillin) encoded by *blaZ* gene. In the late 1950s, methicillin (an antibiotic that cannot be hydrolyzed by β lactamase enzyme) was introduced into human medicine. However, shortly after their introduction, methicillin‐resistant *S. aureus* (MRSA) strains were reported. The *mecA* gene is the gene that causes methicillin resistance. This gene encodes another penicillin binding protein, called PBP2A, which has a low affinity for β lactam antibiotics. The *mecA* gene is part of a large mobile genetic element called the Staphylococcal cassette chromosome *mec* (SCCmec). MRSA is usually resistant to multiple drugs (Lee, 2003; Robinson & Enright, 2003).

MRSA has been increasingly reported as a new problem in veterinary medicine. The appearance of MRSA causes serious zoonotic diseases (Vanderhaeghen et al., 2010). It was first described as a hospital‐based cause of infection, but it has received attention as a community pathogen (Said‐Salim et al., 2003). It has been isolated from cattle, dogs, cats, pigs, horses and poultry all over the world (Leonard & Markey, 2008).

Therefore, the objectives of this study are to determine the occurrence of MRSA and determine the drug sensitivity pattern of MRSA.

## 2 MATERIALS AND METHODS

### 2.1 Study area

Adama (Nazret) is one of the largest towns in Oromia region of Ethiopia. It is about 100 km away from Addis Ababa in the southeast direction at an altitude of 1650 m above sea level. Its annual temperature ranges from 13.9–29°C. It is located at 8^°^33´35´´N‐‐8 ^°^ 36´46´´N latitude and 39 ^°^ 11´57´´E‐‐39 ^°^ 21´15´´E longitude. The total population of Adama district is 155, 321 of which, 76,325(49.14%) were females and 78,996(50.86%) were males according to the 2007 population and housing census of Ethiopia (CSA, 2008).

### 2.2 Study population

The target dairy population is 50% local* Holstein cows with different age, lactation stage, feeding conditions and hygiene milking status. Cows were selected randomly from the selected 15 large and 10 small dairy farms in and around Adama town. The age of the individual animals was determined based on their dentition. Breeds of animals were distinguished by their uniquely observable characteristics and body condition score was made and recorded as poor, medium and good according to Nicolson and Butterworth (1986).

### 2.3 Sample size determination

The sample size required was determined, according to Thrusfield (2007), with defined precision of 5% and level of confidence of 95%.

$$N=\frac{\;1.96^{2\;}\mathrm{Pexp}\;\left(1-\mathrm{Pex}\right)}{d^{2}}$$

where *N* is the required sample size, *d* is the desired absolute precision, Pexp is the expected prevalence, the previous prevalence in the area.

According to the formula, when there is no prior study in the area, it should be conducted that taking Pexp 0.5 for larger sample size, the calculated sample size was 384 lactating cows from the conveniently selected dairy farms in the study areas.

### 2.4 Study design

A cross‐sectional study was conducted starting from October 2015 to June 2016.

### 2.5 Sampling techniques and data collection

#### 2.5.1 Milk sample collection

Twenty‐five dairy farms were selected randomly using list of farms in and around Adama as a sampling frame and lactating cows were selected randomly from the lists obtained from each selected farm. Clinical mastitis was recognized by visual inspection and palpation of the udder for injuries, pain, heat and swelling. In addition, the milk of each quarter was withdrawn, confirmed the change of colour and consistency. A California mastitis test was performed. Mild circular movement was applied to the paddle. The positive samples indicate the gel formation in a few seconds. The results were qualified according to the gel formation and were classified as negative if there was no gel formation. The cows were considered positive if at least a quarter is positive per california mastitis test (CMT) (Radostits et al., 1994). The milk samples were collected before milking, the teats were washed with tap water, dried and the teats were immersed in a cotton bar with 70% ethyl alcohol. After discarding the first three milking flows, the milk was recovered from the cows’ aseptically (NMC, 1990). The samples were transported to the microbiology laboratory, Addis Ababa University, and stored at 4°C to the culture of standard bacteriological media. A California mastitis test was performed.

#### 2.5.2 Culturing procedure

The loopful milk sample was aseptically spread on a sterile blood agar plate (BAP) (Appendix A) enriched with 7% heparinized sheep blood, and incubated for 24–48 h at 37°C under aerobic culture conditions. Then it was checked for the presence of staphylococcal colonies in the plate. The isolates allegedly belonging to the genus Staphylococcus were collected according to their morphological characteristics (milky white, grey, white or yellow colonies) and the haemolysis pattern on the BAP surface. The putative staphylococcal colonies were then sub‐cultured on nutrient agar plates (NAP) that contains lab‐lemco powder 1.0, yeast extract 2.0, pepton 5.0, sodium chloride and incubated at 37°C for 24–48 h to obtain pure cultures (Quinn et al., 1999).

#### 2.5.3 Isolation and identification of *S. aureus*

Final identification of staphylococci organisms and species assignment were done based on Gram staining, catalase test, sugar fermentation (Appendix B) and coagulase test (Mekonen, 2009).

##### Gram's staining

All cultures of suspected Staphylococcus were Gram stained and observed under an optical microscope to determine the Gram response, cell size, shape and arrangement. Gram‐stained smears showing typical colonies of Gram positive cocci in irregular grape‐like clusters are regarded as putative staphylococcal species (Quinn et al., 2002).

##### Catalase test

The pure culture was collected using a sterile loop and mixed with a drop of 3% H_2_0_2_ on a clean glass slide. If the organism is positive, oxygen bubbles are released within a few seconds, while catalase negative isolates do not produce bubbles. Catalase positive cocci were considered as Staphylococcus (Quinn et al., 2002).

##### Oxidation and fermentation test

The oxidation and fermentation (OF) medium containing glucose is green. If the bacteria produce acid, the medium will turn yellow fermenting the glucose. Bacteria that can metabolize glucose under aerobic or anaerobic conditions are called facultative anaerobes, which are considered as Staphylococcus (Quinn et al., 1999).

##### Mannitol salt agar (MSA)

Colonies identified as Staphylococcus by the Gram stain reaction, catalase test, and OF test were streaked onto mannitol salt agar (MSA) plates and incubated at 37°C, and growth and colour changes of the medium were checked 24–48 h afterwards. Changes in growth and pH (red to yellow) in the medium are considered confirmatory identification of *S. aures*. Fermentation of mannitol by *S. aureus* causes yellowing of the medium (Quinn et al., 2002).

##### Coagulase test

A test tube coagulase test was performed in a sterile test tube by adding 0.5 ml of selected *S. aures* isolates, grown in Tryptone Soy Broth (TSB) for 24 h at 37°C, to 0.5 ml of citrated rabbit plasma, then gently swirling to mix and incubate the tube with a negative control tube containing a mixture of 0.5 ml sterile TSB and 0.5 ml rabbit plasma at 37°C. Coagulation was assessed after the first 4 h of the test and the following 24 h of incubation. If any degree of solidification from loose clots to immovable solid clots was seen in the test tube, when the tube was inverted (tilted), the reaction was considered positive, and no degree of solidification were considered negative (Appendix C) (Quinn et al.,^1999^)

##### Purple agar base

Purple agar medium (PAB) supplemented with 1% maltose is used to distinguish types of pathogenic staphylococci, especially coagulase positive isolates. The suspect culture was inoculated ono a plate of PAB medium containing 1% maltose and incubated at 37°C for 24–48 h. The identification is based on the fact that the rapid fermentation of maltose and acid metabolites by *S. aureus* causes the pH indicator (bromocresol purple) to turn the culture medium and colonies to yellow (Quinn et al., 2002).

#### 2.5.4 Identification of methicillin‐resistant *S. aureus*

Using the aforementioned bacteriological method, *S. aureus* strains were isolated from milk samples from the mastitis positive cows in the study area. The Kirby–Bauer disk diffusion antibiotic susceptibility method was used to detect methicillin resistance. The oxacillin disc was used to detect methicillin‐resistant *S. aureus* strains. Briefly, a single colony was suspended in 1 ml of saline, adjusted to the 0.5 McFarland standards, and spread evenly over the surface of the agar plate with a sterile cotton swab. After the agar plate surface was dried for approximately 5 min, it was incubated with 1 μg oxacillin antibiotic disc on Mueller–Hinton agar plates at 35°C for 16–24 h and then the result was obtained. The results are explained by measuring the diameter of the area with the help of a sliding calliper, including the diameter of the disc (CLSI, 2009).

#### 2.5.5 Determination of drug susceptibility of MRSA

Only MRSA strains were tested for their susceptibility to different antibiotics. Streptomycin (Strep, 10 μg), amoxicillin (Am, 25 μg), kanamycin (k, 30 μg), nalidixic acid (NA, 30 μg), oxytetracycline (OT, 30 μg), sulfamethoxazole (S, 300 μg) and ceftriazole (CRO, 30 μg) discs were used to determine antimicrobial susceptibility. It was performed according to CLSI (2009) guidelines using the Kirby–Bauer disk diffusion antibiotic susceptibility method (Appendix D).

### 2.6 Data analysis

All collected data on race, age, stage of lactation, milking hygiene, type of dairy farm and test results were recorded by interviewing farm owners or attendants and observation. The complete data were entered and coded to Microsoft office spread sheet then transferred to the SPSS 20 software program on the computer before analysis. All measurement parameters were analysed using SPSS 20 statistical software. The chi‐square test was used to compare categorical variables, and the significance level was determined at the 95% confidence level (*p* < 0.05).

#### 2.6.1 RESULTS

Out of 384 animals included in this particular study, the prevalence of mastitis was 121(31.5%). Among them, 37 cases (30.6%) were clinical mastitis, and 84 cases (69.4%) were subclinical mastitis. Milk samples from 121 CMT positive cows were subjected to microbiological examination and 37(30.6%) (Table 1) *S. aureus* strains were isolated. The results showed that the prevalence of mastitis in the study area was significantly affected by internal and external factors. There are significant differences in the prevalence of mastitis in different age groups (*p* < 0.05) and races (*p* < 0.05), and the prevalence of mastitis in the late period of lactation increased significantly (*p* < 0.05). Management factors such as milker hygiene (*p* < 0.05) and floor type (*p* < 0.05) were external risk factors that significantly affect the prevalence of bovine mastitis. In general terms, the prevalence of mastitis was significantly higher in the following populations; cross breed cows (43.5%), old cows > 7 years (51.2%), late lactation (39.9%), muddy soil type (40.9%)) and farms with poor milker hygiene (49.2 %) (Tables 2 and 3).

**TABLE 1:** Number and percentage of *S. aureus* isolated from clinical cases and CMT positive subclinical cows

Forms of mastitis	No. examined	No. of *S. aureus* isolate	Prevalence of *S. aureus*
Clinical	37	5	13.5%
Sub clinical	84	32	38.1%
Total	121	37	30.6%

**TABLE 2:** The association of potential intrinsic risk factors with mastitis (*n* = 384)

Risk factors	No. examined	Affected number	Prevalence%	*X* ^2^ (*p*‐Value)
Breed				
Boran	170	28	16.5	31.97 (0.00)
HF cross breed	214	93	43.5	
Age				
<4	137	27	19.7	
4–7	206	73	35.4	17.69 (0.00)
>7	41	21	51.2	
Lactation stage				
<3	79	19	24.1	4.67 (0.09)
3–6	222	69	31.1	(0.09)
>6	83	33	39.8	(0.03)

**TABLE 3:** The association of potential extrinsic risk factors with mastitis (*n* = 384)

Risk factors	No. examined	Affected number	Prevalence %	X^2^ (*p*‐Value)
Floor type				
Muddy	220	90	40.9	21.085 (0.00)
Concrete	164	31	18.9	
Hand washing				
No	185	91	49.2	51.698 (0.00)
Yes	199	30	15.1	

From the total of 37 *S. aureus* isolates, 12(32.4%) isolates were identified as methicillin‐resistant *S. aureus* strains.

Regarding to drug susceptibility test, MRSA strains were highly resistant to amoxicillin (75%), oxytetracycline (66.7%). Next to amoxicillin MRSA isolates were resistant to oxytetracycline (66.7%) (table 4).

**TABLE 4:** Antimicrobial sensitivity profile MRSA isolated from dairy cow's milk in and around Adama (*n* = 12)

Antimicrobial	Susceptible No. (%)	Intermediate No. (%)	Resistance No. (%)
Kanamycin	9(75)	3(25)	0(0)
Ceftriazole	5(41.6)	3(25)	4(33.3)
Oxytetracycline	2(16.6)	2(16.6)	8(66.7)
Streptomycin	7 (58.3)	1(8.3)	4(33.3)
Nalidixic acid	6(50)	1(8.3)	5(41.6)
Sulphonamied	4(33.3)	2(16.6)	6(50)
Amoxicillin	3(25)	0(0)	9(75)

*Note*: Percentage was calculated by dividing the number confirmed as susceptible, intermediate and resistant in each disk by the total number of MRSA isolate.

## 3 DISCUSSION

Mastitis in the cattle reported in this study (31.5%) was consistent with the early reports of Bekele et al. (2012) who reported a prevalence of 34.3%. However, it was lower than Abera et al. (2010) and Hundera et al. (2005) who reported a prevalence of 46.7% and 52.8% around Adama and Sebeta, respectively. The variability of the prevalence of mastitis between the different reports might be due to differences in farm management practices and research methods that are adopted and differences in the equipment. The prevalence of the subclinical mastitis in this study was 69.4%. It was relatively closer with Zeryehun et al. (2013) and Nibrat et al. (2011) who reported 51.5% and 56% prevalence. The reported prevalence was higher than the report of 36.67% by Hundera et al. (2005), 38.2% by Workneh et al. (2002) and 22.3% by Getahun et al. (2008). In this study, the general prevalence of clinical mastitis was less than that of subclinical mastitis. Similarly, Bekele et al. (2012) and Workneh et al. (2002) indicated the higher prevalence of subclinical mastitis than clinical mastitis. The variation of morbidity between the sub‐clinical and clinical mastitis may be due to the fact that the cow's defence mechanism reduces the severity of the disease. In, Ethiopia, nonclinical mastitis has been ignored and strives are made to treat clinical cases (Kerro & Tareke, 2003).

Thirty‐seven isolates (30.6 %) of *S. aureus* were identified with bacterial testing of 121 samples of milk. This result was comparable to Belayneh et al. (2013) (32%) and Zingeser et al. (1991) (27%). The current findings were lower than Hundera et al. (2005) (44.4%). However, it was higher than the reports made by Hussein (1999) (10.6%). Relative prevalence of *S. aureus* might be related to ineffective udder and hand washing, the lack of hand washing, the use of dirty clothes for teat disinfection and milking materials disinfection. The high incidence rate of *S. aureus* could also be due to the wide spread of organisms in the mammary gland, allowing it to survive in the udder and establish chronic and low‐level clinical infections (Radostits et al., 1994).

MRSA is recognized as one of the main causes of hospital infection and it is also responsible for a wide range of infections (Gould et al., 2012; Wang et al., 2012). The results of this study showed that 32.4% of the isolates were MRSA. This result is lower than those reported by Daka et al. (2012) (60.3%) in Sothern Ethiopia. However, this result is consistent with what Mark et al. (2003) (34.7%) reported in France. The possible description of methicillin resistance is because of the *mecA* gene and the presence or overexpression of β‐lactamase enzymes and chromosomal variation (Banerjee et al., 2010; Gal et al., 2001). The difference in antimicrobial susceptibility to drugs may reflect the different antibacterial use and the use of inappropriate antibiotics in different areas. The choice of antibiotics for the treatment of MRSA infection is generally complicated. In this study, there was a high proportion of MRSA isolates (75%) resistant to amoxicillin, this agrees with (Mollering, 2012). As a result, penicillin and other beta‐lactam antibiotics cannot be used to treat MRSA infection. The most important resistant mechanism for β‐lactamase antibiotic is the production of beta‐lactamase that inactivates β‐lactams (e.g., amoxicillin) by hydrolysing the beta‐lactam ring. In the present study, amoxicillin disc diffusion zone of the edge test was adopted to detect β‐lactamase production, and most MRSA isolates were positive for β‐lactamase production.

**FIGURE 1:**
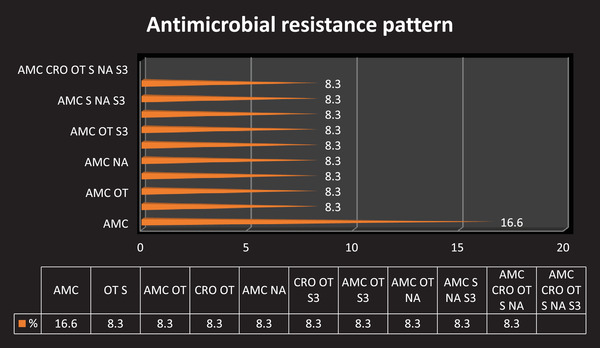
Antimicrobial resistance pattern of MRSA isolates. **Note**: The percentages of the phenotypes were calculated by dividing the number of the particular phenotype by the total number of antibiotic‐resistant isolates identified in a given area. Of the 12 MRSA isolates five of them show resistance to more than two drugs (total MDR = 0.41.65%)

This is due to the indiscriminate use of antibiotics/antimicrobial agents for prevention and treatment purposes. In our study, most MRSA strains are multidrug resistant (Figure 1). These results are consistent with the overall findings of Kesah et al., 2003, that show MRSA strains are multidrug resistant (Peng et al., 2010; ).

## 4 CONCLUSION AND RECOMMENDATIONS

Increasingly, MRSA has been isolated from bovine mastitis. Therefore, *S. aureus* infection remains one of the major public health problems in and around Adama. The abuse of antibiotics / antimicrobial agents for prevention and other therapeutic purposes may be the reason for the increased resistance of *S. aureus* to antimicrobial drugs. A relatively large number of strains are resistant to antibiotics commonly used in the treatment of mastitis. In addition, antimicrobial susceptibility indicates that MRSA isolates are more resistant to β‐lactam antibiotics. Therefore, the emergence of MRSA poses a serious threat to livestock and public health.

Based on the above concluding remarks, the following recommendations are proposed:

- Antimicrobial susceptibility test should be carried out at a regular interval with a view to selecting appropriate therapy is needed.
- Proper hygienic and improved managemental practices should be introduced at farm level.
- Creating public awareness about transmission, prevention and control of MRSA is paramount important.
- Future studies should focus on determining the antimicrobial resistance mechanisms MRSA isolates to fix the problem.

## CONFLICT OF INTEREST

The authors declare no conflict of interest.

## AUTHOR CONTRIBUTIONS

Muhaba Yimana: Data curation, Formal analysis, Investigation, Methodology, Writing original draft, Writing review & editing. Juhar Bekele: Data curation, Methodology, Validation, Visualization, Writing review & editing.

## ETHIC STATEMENT

The authors confirm that the ethical policies of the journal, as noted on the journal's author guidelines page, have been adhered to and the appropriate ethical review committee approval has been received.

### PEER REVIEW

The peer review history for this article is available at https://publons.com/publon/10.1002/vms3.902

## Data Availability

The data supporting the present result will be available up on request from the first author.

## APPENDIX A

### MEDIUM USED FOR BACTERIAL IDENTIFICATION

#### I Blood agar base (Oxoid, England)

Composition (g/L): Hear muscle, infustion from(solid) 2.0; pancreatic digest of casein 13.0; yeast extract 5.0; sodium chloride 5.0; agar 15.0.

Direction: Suspend 40 g of powder in 1 L of distilled water. Mix throughly and heat with frequent agitation and boil for 1 min to completely disolve the powder. Autoclave at 121°C for 15 min. Cool the base to 45–50°C and 5%–7% sertile defiberinated sheep plood.

#### II Grams reagent

‐ Crystal violet
‐ Iodine (mordant)
‐ Ethanol 95%
‐ counter‐stain(sufranin)

#### III Nutrient agar (Oxoid, England)

Composition(g/L): Lab‐lemco powder 1.0; yeast extract 2.0; pepton 5.0; sodium chloride 5.0; Agar 15; pH:7.4 ± 0.2.

Direction: Suspend 28 g in 1 L of distilled water. Boil to completely dissolve the powder. Sterilize by autoclaving at 121°C for 15 min.

#### IV O‐F basal medium (Himedium, India)

Composition (g/L): Sodium chloride 5.0; casein enzymatic hydrolysate 2 g; agar 2 g; dipotassium phosphate 0.3 g; bromothymol blue 0.08 g.

Direction: Dissolve 9.4 g in 1000 ml distilled water. Gently heat to dissolve the medium completely. Sterilize by autoclaving at 121°C for 15 min. Add 10% sterile glucose solution. Mix and dispense in 5 ml amounts in sterile tube in duplicate for aerobic and anearobic fermentation solution.

#### V Mannitol salt agar (Oxoid, England)

Composition (g/L): Lab‐lemco poweder 1.0; peptone 10.0; mannitol 10.0; sodium chloride 75.0; phenol red 0.025; agar 15.0; pH 7.5.

Direction: Suspend 111 g in 1 L of distilled water. Boil to completely dissolve the powder, and sterilze by autoclaving at 121°C for 15 min.

#### VI Purple agar base

Composition (g/L): Peptone, special 10.00; beef extract 1.00; sodium chloride 5.00; bromocresol purple 0.020; agar 15.00; final pH (at 25°C) 6.8 ± 0.2.

Direction: Suspend 31.02 g in 1000 ml distilled water. Add 5–10 g of the carbohydrate to be tested. Boil to dissolve the medium completely. Dispense in tubes as desired and sterilize by autoclaving at 15 lbs pressure (121°C) for 15 min. Alternatively sterilize the basal medium prepared using 900 ml distilled water, and add 100 ml separately sterilized 5–10% solution of the desired carbohydrate to it.

#### VII Muller Hinton agar (Oxoid, England)

Composition (g/L): Beef extracts 2; acid hydrolysate of casein 17.5; starch 1.5; agar 17.
Direction: Suspend 38 g of the powder in 1 L of distilled water. Mix throughly, heat with frequent agitation and boil for 1 min to completely dissolve the powder; autoclave at 121°C for 15 min.

#### VIII Tryptone soya broth (Oxoid, England)

Composition (g/L): Pancreatic digest of casein 17; papaic digest of soybean meal 3; sodium chloride 5; di‐basic potassium phosphate 3.5; glucose 2.5; final pH = 7.3 ± 00.2 at 25°C final containers.
Direction: Dissolve 30 g in 1 L of distilled water and sterilize by autoclaving at 121°C for 15 min.

### APPENDIX B: PRIMARY BIOCHEMICAL TESTS

#### I Gram's staining procedure

1. Make a thin bacterial colony smear and allow it to dry in air.
2. Fix the dried smear by passing through the Bunsen flame two to three times.
3. Flood the slide with crystal violet (primary stain) for 60 s and wash with tap water.
4. Flood the slide with Gram's iodine solution for 60 s and wash with tap water.
5. Flood the slide with 95% ethanol alcohol for 5–10 s and wash with tap water.
6. Finally, flood with carbol fuschin (counter stain) for 60 s and dry in air.

#### II Procedure for catalase test

1. Place a drop of 3% H_2_O_2_ on a glass slide.
2. Touch a sterile loop to a culture of the organism to be tested and pick up a visible mass of cells (colony).
3. Mix the organism in the drop of hydrogen peroxide.
4. Observe for immediate and vigorous bubbling. Interpretation: Bubbling indicates a positive test and no bubbling indicates a negative test.

#### III Procedures for oxidation and fermentation test

1. Prepare O‐F base medium and when the O‐F base cooled at 50°C, add 20 ml sterile glucose solution into 200 ml of O‐F base for a final concentration of 1% glucose, and dispense into tubes.
2. Two tubes of O‐F medium are heated in a beaker of boiling water immediately before use to drive off any dissolved oxygen and the tubes are then cooled rapidly under cold running water.
3. Both tubes are stab inoculated with the bacterium and a layer of sterile paraffin oil is layered on top of one of the incubated at 37°C and examined in 24 h and then daily for up to 14 days.

### APPENDIX C: SECONDARY BIOCHEMICAL TESTS

#### I Procedure for coagulase test

1. Using a sterile pipette, add 0.5 ml of the rehydrated plasma to a 12 × 75 mm test tube.
2. Using a sterile serological pipette, add 0.5 ml of the overnight broth culture of the test organisms to the tube of plasma or, using a sterile bacteriological loop, thoroughly emulsify two to four colonies (one loopful) from a no inhibitory agar plate in the tube of plasma.
3. Mix gently and incubate at 37°C.
4. Examine periodically for coagulation by gently tipping the tube after the first hour and once every hour thereafter until 4 h have elapsed. If no clot is observed at the end of this period, examine at 24 h. Avoid shaking or agitating the tube during reading. Doubtful or false‐negative results may occur due to breakdown of the clot.
5. Record results: Positive for any degree of clotting—from a loose clot suspended in plasma to a solid clot that is immovable when the tube is inverted, and negative for no degree of clotting.

## APPENDIX D ANTIMICROBIAL SUSCEPTIBILITY DIAMETER ZONE FOR STAPHYLOCOCCAL ISOLATES

**Table:**

Antimicrobial agent	Potency	Zone diameter nearest whole number,Mm		
		*S*	*I*	*R*
Kanamycin	30	≥18	14**–**17	≤13
Ceftriazole	30	≥21	14–20	≤13
Oxytetracycline	30	≥28	‐	≤19
Streptomycin	10	≥15	12–14	≤11
Nalidixic acid	30	≥19	14–18	≤13
Sulphonamied	300	≥17	13**–**16	≤12
Oxacillin	1	≥13	11–12	≤13
Amoxicillin	25	≥20	‐	≤19
